# Macrophage polarization regulates intervertebral disc degeneration by modulating cell proliferation, inflammation mediator secretion, and extracellular matrix metabolism

**DOI:** 10.3389/fimmu.2022.922173

**Published:** 2022-08-18

**Authors:** Xiao-Chuan Li, Shao-Jian Luo, Wu Fan, Tian-Li Zhou, Dan-Qin Tan, Rong-Xiong Tan, Qun-Ze Xian, Jian Li, Chun-Ming Huang, Mao-Sheng Wang

**Affiliations:** ^1^ Postdoctoral Innovation Practice Base of Gaozhou People’s Hospital, Gaozhou, China; ^2^ Department of Orthopedic Surgery, Gaozhou People’s Hospital, Gaozhou, China; ^3^ Central Laboratory of Orthopedics, Gaozhou People’s Hospital, Gaozhou, China

**Keywords:** low back pain, intervertebral disc degeneration (IDD), inflammation, nucleus pulposus cells (NPCs), macrophage polarization, musculoskeletal disorder

## Abstract

Macrophage infiltration and polarization have been increasingly observed in intervertebral disc (IVD) degeneration (IDD). However, their biological roles in IDD are still unrevealed. We harvested conditioned media (CM) derived from a spectrum of macrophages induced from THP-1 cells, and examined how they affect nucleus pulposus cells (NPCs) *in vitro*, by studying cell proliferation, extracellular matrix (ECM) synthesis, and pro-inflammation expression; and *in vivo* by injection CM in a rat IDD model. Then, high-throughput sequencing was used to detect differentially expressed genes (DEGs). Gene Ontology (GO), the Kyoto Encyclopedia of Genes and Genomes (KEGG), and protein-protein interaction (PPI) networks were used to further analysis. Higher CCR7+ (M1 marker) and CD206+ (M2 marker) cell counts were found in the degenerated human IVD tissues as compared with the control. Furthermore, the cell co-culture model showed M1CM attenuated NPC proliferation, downregulated the expression of ECM anabolic genes encoding aggrecan and collagen IIα1, upregulated the expression of ECM catabolic genes encoding MMP-13, and inflammation-related genes encoding IL-1β, IL-6, and IL-12, while M2CM showed contrasting trends. In IDD model, higher histological scores and lower disc height index were found following M1CM treatment, while M2CM exhibited opposite results. M1CM injection decreased ECM anabolic and increased ECM catabolic, as well as the upregulation of inflammation-related genes after 8 weeks treatment, while M2CM slowed down these trends. Finally, a total of 637 upregulated and 655 downregulated genes were detected in M1CM treated NPCs, and 975 upregulated genes and 930 downregulated genes in the M2CM groups. The top 30 GO terms were shown and the most significant KEGG pathway was cell cycle in both groups. Based on the PPI analysis, the five most significant hub genes were PLK1, KIF20A, RRM2, CDC20, and UBE2C in the M1CM groups and RRM2, CCNB1, CDC20, PLK1, and UBE2C in the M2CM groups. In conclusion, macrophage polarization exhibited diverse roles in IDD progression, with M1CM exacerbating cell proliferation suppression and IVD degeneration, while M2CM attenuated IDD development. These findings may facilitate the further elucidation of the role of macrophage polarization in IDD, and provide novel insights into the therapeutic potential of macrophages.

## Introduction

Low back pain (LBP) is one of the most common musculoskeletal disorders worldwide, and it has been estimated that more than 70% of the global population suffers from this disorder in their lives (1). Current treatments for LBP can alleviate pain to some extent; however, they do not target the underlying causes. Furthermore, surgical treatments, such as disc decompression or fusion may cause complications, including adjacent segment degeneration and spinal mobility loss (2). It is urgently needed efficient therapeutic strategies for restoring the biological function of the intervertebral disc (IVD) or retarding or reversing intervertebral disc degeneration (IDD) (3). The IDD process is associated with a shift in the homeostatic balance between anabolism and catabolism; thus, it results in a decreased secretion of extracellular matrix (ECM) components, particularly aggrecan and collagen IIα1 (4), and an enhanced expression of matrix metalloproteinases (MMPs), which are the key matrix-degrading proteases involved in IDD progression (5). Additionally, the progression of IDD is accompanied by significant increases in the levels of various pro-inflammatory cytokines, including interleukin (IL)-1α IL-6, IL-8, and IL-12 and tumor necrosis factor (TNF)-α (6). Hence, it is critical to promote the secretion of ECM components as well as anti-inflammatory responses to combat IDD. Meanwhile, to investigate the significant molecular and key pathways, which suggest functional importance in IDD is necessary to clarify the pathophysiology and molecular mechanisms, may supply potential therapeutic targets.

Immune homeostasis maintained by variety immune cells is believed to play a vital role in the IDD process, including T and B cells, mast cells, and macrophages (7). Macrophages have recently attracted considerable attention in the process of IDD, due to their ability in keeping the homeostasis of heart (8), skeletal muscle (9), skin (10), and spinal cord (11). In IDD, as the immunologic balance is damaged by annulus rupture or end plate micro-fractures, macrophage infiltration and polarization occur (12). An increasing number of cell co-cultured models have found nucleus pulposus cells (NPCs) and macrophages could change inflammatory factors expression, such as inflammation-related genes (*IL-1β*, *IL-6*, and *Ccl3*) and ECM metabolism-related genes (*MMP13* and *Acan*) in both cells (13, 14). Macrophages are classified as activated M1 macrophages or activated M2 macrophages based on their responses to microenvironmental stimuli (15), and both were found in human IVDs recently (16). However, the role of macrophage polarizations on NPCs and their potential mechanism in IDD remain unrevealed.

In this study, we investigated the expression of macrophage polarization markers in human IDD samples, and then harvested conditioned media (CM) from a spectrum of macrophages. We tested their biological effects on NPCs in cell co-culture model, and we also conducted *in vivo* experiments by injecting the CM into rat coccygeal IDD models. Finally, we used high-throughput sequencing to identify differentially gene expression genes (DEGs) and performed Gene Ontology (GO) and Kyoto Encyclopedia of Genes and Genomes (KEGG) pathway analysis. Our aim was to explore the biological role of macrophage polarizations and explore potential molecular targets and signaling pathways associated with IDD. This may enhance understanding regarding the cytological mechanism of IDD pathogenesis and lead to the development of novel cell regenerative therapies for IDD treatment, particularly for patients in the early stages of the disease.

## Materials and methods

### Clinical sample collection and ethics statement

The clinical and animal study was reviewed and approved by the Ethics Committee of Gaozhou people’s Hospital, Guangdong, China (No. 2019-039). Human NP tissues were isolated from 13 patients and detailed information regarding the patients is presented in **Table S1**. In addition, human NPCs were isolated and cultured from eight patients and the detailed information is presented in **Table S2**. Written informed consent was obtained from the individual(s) and/or minor(s)’ legal guardian/next of kin for the publication of any potentially identifiable images or data included in this article. The study was performed according to the amended declaration of Helsinki.

### Human NP specimen collection and immunohistochemistry

Human NP tissues were harvested and fixed with 4% paraformaldehyde within 30 min. After 12 h, the NP tissues were washed three times with phosphate-buffered saline (PBS), and then dehydrated and embedded in paraffin. Finally, 5-µm thick serial sections were cut and stored at 4°C for further study. For immunohistochemistry tests, the NP tissue sections were deparaffinized, rehydrated, and soaked in citric acid antigen repair solution buffer (10 mM citric acid, pH 6.0) overnight at 60°C, to expose antigenic epitope. The samples were then soaked in 3% hydrogen peroxide for 15 min to inactivate endogenous peroxidase, and then washed with PBS three times. The sections were blocked with goat serum at room temperature for 30 min, and incubated with primary antibodies CCR7 (1:200, ab89064, Abcam, Cambridge, UK), and CD206 (1:200, ab64693, Abcam, Cambridge, UK) overnight at 4°C. Finally, the sections were stained with horseradish peroxidase-conjugated secondary antibodies (Jackson ImmunoResearch Laboratories, West Grove, PA, USA), and then 3, 3-diaminobenzidine was used to visualize the chromogen with hematoxylin used for counterstaining. After dehydration and clearing with dimethylbenzene, the sections were sealed with neutral gum, and images were obtained using a Leica inversion microscope (Leica, Wetzlar, Germany). For marker expression analysis, 20× magnification bright-field microscopy (Olympus, Tokyo, Japan) was used for each specimen, and five fields that included unhealthy regions and macrophage accumulation were selected throughout the entire IVD section. Unhealthy regions were defined as areas with extensive damage and obvious defects in ECM organization and cellular distribution patterns (i.e., granulation-like tissue, tears, cracks, ruptures, cell clustering, sclerosis, and irregular contours or decreased thickness of the endplate) (12). If no cells tested positive for the markers or unhealthy regions in the field of vision, the microscope position was moved to another chosen area. The total number of cells in each IVD was counted in consensus by two authors (TDQ and ZTL) using ImageJ (U.S. National Institutes of Health, Bethesda, MD, USA). The number of positively stained NP macrophages was calculated as the mean value obtained from the five fields analyzed.

### Animals and surgically induced IDD model

Sixty 2-month-old male Sprague-Dawley rats were purchased from the Guangdong Province Experimental Animal Center (Guangzhou, China). The animals were kept in a ventilated environment with a 12:12-h light–dark cycle at a constant temperature of 22°C. The rat coccygeal IDD model was established by needle puncture as previously described (17). Briefly, the animals were anesthetized by intraperitoneal injection (7 µL/g) of 10% chloral hydrate (Sigma, USA), and the experimental model of IDD was established by percutaneous IVD puncture with a 20-gauge needle on levels 7–8 (Co7–8) and 8–9 (Co8–9) of the rat coccygeal IVD. Thereafter, the needle was rotated 360°and held for 30 s, and then removed. In the study, we selected Co7–8 as the different type CM treatment group (n = 18 in each group), Co8–9 IVD as the 1640 culture medium negative control (NC) group, and non-punctured IVD at Co9–10 as the blank control (BC) group. The Co7–8 segment was divided into three groups, which were randomly treated with M2CM, M1CM, and M0CM. Finally, 10 μLCM of the different groups were injected into the center space of the NP at a depth of 6 mm. All injections were performed with a 33 gauge Hamilton syringe (Hamilton, USA), and this needle size did not induce degenerative disc changes. We applied several methods to minimize the difference variation and error in the IDD model: i) The needle puncture in the acupuncture model was limited to the depth of 6 mm to ensure a consistent puncture depth; ii) the puncture segments of coccygeal IVDs (Co7–8 and Co8–9) were previously located and marked *via* X-ray monitoring; iii) M2CM injection was performed using a micro injector that is widely used in drug administration; iv) all the operations were performed by the same team member.

### Isolation and cultivation of human NPCs

NPCs were harvested as previously described (18). Firstly, NP tissues were collected and immediately transported to a cell culture room under sterile conditions. The NP tissues were washed at least three times with PBS to remove blood and mechanically minced into small pieces (<1 mm^3^). Then, 0.2% collagenase II (Sigma, USA) was used to digest NP tissues for 4 h at 37°C in a humidified incubator. Finally, the suspended cells were filtered with a 100-μm mesh filter and centrifuged at 100 × *g* for 5 min, which was followed by two washes with PBS. Finally, the cell pellets were re-suspended and cultured in standard culture medium, consisting of F12 DMEM (HyClone), 10% fetal calf serum (Gibco), and 1% penicillin/streptomycin (Gibco) in 25-cm^2^ cell culture flasks at a density of 1 × 10^5^ cells/mL; cells were cultured in a humidified incubator at 37°C under 5% CO_2_. After 2 days, the suspended cells and medium were half changed, and the adherent cells were cultured and expanded by completely replacing the medium every 2–3 days. As the cells reached 70–80% confluency, the primary cells were harvested and passaged. Passage 1 (P1) NPCs were harvested with 0.25% trypsin-ethylenediaminetetraacetic acid (EDTA; Sigma) for 1 min and subcultured at a ratio of 1:3. The culture medium was refreshed every 1-2 h to remove floating cells. P3-6 cells were harvested for identification and subsequent experiments.

### Macrophage polarization induction and conditioned medium collection

**Figure 1:**
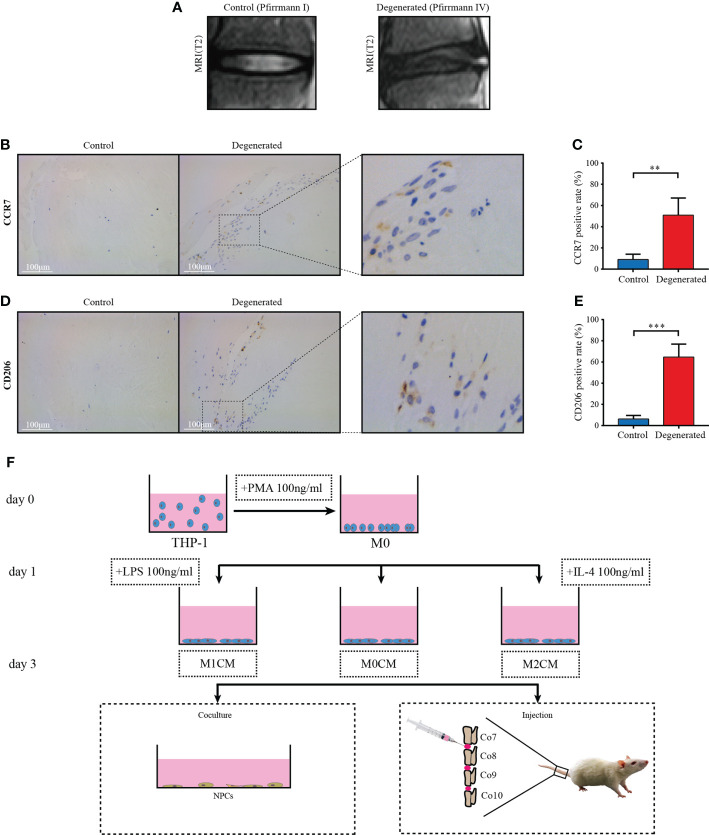
Macrophage accumulation and polarization in human IVD tissues. **(A)** Representative MR images showing normal (Pfirrmann grade I, n = 5) and IDD samples (Pfirrmann grades III-V, n = 8), respectively. **(B)** Representative images showing the immunolocalization of CCR7-positive cells in human normal IVD tissues; **(C)** Comparison of the proportion of positive cells corresponding to normal and degenerated IVD samples. **(D)** Representative images showing the immunolocalization of CD206-positive cells in human IDD tissues, and **(E)** Comparison of positive cells corresponding to normal and IDD samples. The data were analyzed using Mann–Whiney U test. **p < 0.01; ***p < 0.001. Data are shown as mean ± SD. IVD, intervertebral disc; IDD, intervertebral disc degeneration. **(F)** Flow diagram of harvesting conditioned medium (CM) and grouping process of CM *in vitro* and *in vivo*.

Human THP-1 monocytes were induced to differentiate into M0, M1, and M2 macrophages as previously described (5, 19). Briefly, cells were seeded at a density of 2×10^6^ in175-cm^2^ cell culture bottles for 24h, and then treated with 100 ng/mL PMA (Sigma, USA) for 24h to achieve M0 polarization. Thereafter, cells were transferred into serum-free media and treated for another 24h with 100 ng/ml LPS (Sigma, USA) to achieve M1 polarization and with 50 ng/mL IL-4 (R&D Systems) to promote M2 polarization. The supernatant medium was replaced with serum-free medium and cultured for an additional 24h. Finally, the corresponding supernatant CM was obtained and centrifuged for 15 min at 4°C at 600× *g* to remove cellular debris. This step was repeated at 1500× *g*. The harvested CM was defined as macrophage-derived conditioned media in this study. For the cell co-culture model, NPCs were divided into five groups as follows: group 1 was treated with 10% FBS culture medium as the control, group 2 was treated with 10 ng/mL TNF-α as negative group, and groups 3-5 were treated with 10 ng/mL TNF-α and 30% M0CM, M1CM, and M2CM as the co-culture groups, respectively. A flow diagram of the macrophage-conditioned medium collection is shown in **Figure 1F**.

### Cell proliferation assay

A Cell Counting Kit-8 (CCK-8; Dojindo Laboratories, Japan) was used as previously described to measure cell proliferation (20). Briefly, NPCs were seeded in 96-well plates (2× 10^3^ cells/well), and the three different groups were incubated for 24, 48, 72, and 96 h. After removing the culture medium and CM, 10 μL of CCK-8 solution was added to 100 μL of fresh medium, and the mixture was incubated at 37°C for 1 h. Finally, the samples were analyzed for final measurements. The absorbance of the solution was measured at 450 nm using a microplate absorbance reader (Bio-Rad, USA). A blank 96-well plate was used for zero setting. All experiments were performed four times for each group. For the 5-ethynyl-2’-deoxyuridine (EDU) incorporation assay, cells were cultured in 24-well plates. An EDU incorporation assay was conducted using the Click-iT EDU Alexa Fluor 488 Imaging Kit (Molecular Probes, Thermo Fisher Scientific) according to the manufacturer’s instructions. In brief, cells were incubated with 10 μM EDU and stained with the Click-iT EDU Alexa Fluor 488 Imaging Kit. Cell nuclei were visualized by DAPI. Fluorescence signals were acquired using an Olympus confocal laser scanning microscope (Olympus Corp., Tokyo, Japan). The Image J software 1.8.0 (Media Cybernetics, Silver Spring, MD) was used to calculate the percentage of EDU-positive cells among total cells.

### Total protein isolation and western blotting

Western blotting was performed as previously described (21). Approximately 1× 10^6^ cells were lysed in RIPA buffer (Beyotime) with phenylmethylsulfonyl fluoride (Sigma-Aldrich). The total protein concentration was determined using a BCA protein assay kit (Beyotime). Protein samples (30μg each) were separated through sodium dodecyl sulfate-polyacrylamide gel electrophoresis. The proteins were transferred onto polyvinylidene fluoride membranes (Perkin Elmer). The membranes were blocked with 1xTris-buffered saline and Tween 20 (TBST) containing 5% blotting-grade blocker nonfat dry milk (Bio-Rad). They were incubated overnight with appropriate primary antibodies at 4°C followed by incubation with horseradish peroxidase-conjugated secondary antibody (1:1000; Cell Signaling Technology). Subsequently, the protein bands on the blots were visualized using the Clarity Western ECL Kit (Bio-Rad) and imaged using the ChemiDoc Touch Imaging System (Bio-Rad). The primary antibodies against β-tubulin (#2146) were purchased from Cell Signaling Technology and used at 1:1000 dilution. The primary antibodies against MMP13 (ab51072, 1:1000) and aggrecan (ab3773, 1:100) were obtained from Abcam. Quantification through densitometry of protein bands was performed using the Image-Pro Plus software (Version 5.1; Media Cybernetics, Inc.). The experiment was performed triplicate.

### Quantitative reverse transcription PCR (qPCR) analysis

For NPCs, after incubation under different conditions for 3 days, the total RNA was extracted using TRIzol (Invitrogen) according to the manufacturer’s instructions. For rat coccygeal IVDs, the samples were immediately grinded to a powder in liquid nitrogen, and then, the total RNA was extracted using TRIzol (Invitrogen) according to the manufacturer’s instructions. The RNA was reverse-transcribed using the PrimeScript™ RT Master Mix (TaKaRa, Japan). qPCR was performed in triplicate in 96-well plates, using the SYBR Premix Ex Taq Kit; the final volume of the reaction mixture was 20μL. All primers were obtained from Sangon (Shanghai, China) and are listed in **Table S3**. The qPCR was performed using the One Step SYBR* PrimeScript RT-PCR Kit (TaKaRa, Japan). GAPDH, β-actin, and the 18S gene were used as references, and the average results of three reference genes were used for normalization according to the MIQE-precis guidelines (22). The cycle threshold values were obtained, and data were normalized using the 2^–△△Ct^ method.

### Radiological evaluation

Antero-posterior radiographs of rat coccygeal IVD were taken at 0, 4, and 8 weeks respectively. A fluoroscopic imaging intensifier (radiographs; 48 kV, 10 mA, 60 cm distance) was used, and the digitized radiographic images were stored and evaluated using the picture archiving and communication system (PACS). The measurements included vertebral body height and IVD height. The disc height index (DHI) at each level was determined based on a previous method (23). The DHI was calculated as the percent of the disc height to the length of adjacent vertebral body. The change in the DHI was expressed as %DHI (post-injection DHI/pre-injection DHI). The radiological results of each IVD were performed three times.

### Histological analyses

The IVDs were collected and fixed in 10% formalin for 48 h, decalcified with 10% EDTA (Sigma) for 6 months, and embedded in paraffin wax. The paraffin blocks of IVD were cut into 5 μm coronal sections containing the endplate, annulus fibrosus (AF), and NP, and then stained with either hematoxylin and eosin (H&E) or Safranin-O/Fast green as previously described (17). The samples were examined and photographed using a fluorescence microscope (FV-1000; Olympus). The histological grading scale system included five categories with scores ranging from 0 points (normal) to 15 points (serious degeneration disc) using the method established by Ji et al. (20). The final score was generated by averaging individual scores.

### Immunohistochemical and immunofluorescent analyses

Immunostaining was performed according to the manufacturer’s instructions. To observe the specific expression of collagen type II (ab34712, Abcam) and, aggrecan (13880-1-AP, Proteintech) in the tissues, sections underwent antigen repair with proteases after de-waxing and gradient hydration. Then the tissues were blocked in 5% normal goat serum and incubated with the primary antibodies at 4°C overnight. As a NC, cells or tissues were incubated with isotype IgG control antibodies under similar conditions. After rinsing, the tissue sections were incubated for 1 h with biotin-conjugated secondary antibodies. Color was developed by incubating with the chromogen 3.3’-diaminobenzidine (DAB) tetrahydrochloride, followed by counterstaining with hematoxylin. For immunofluorescent staining, secondary antibodies conjugated with fluorescent tags were added and slides were incubated at room temperature for 1 h in dark. As a NC, cells were incubated with isotype IgG control antibodies under similar conditions. After the cells had been washed, they were incubated with anti-rabbit secondary antibody (Jackson, USA) at a dilution of 1:100 for 1 h at room temperature. Following this, cell nuclei were stained with DAPI solution (1:1000; Invitrogen) for 5 min at room temperature. The samples were examined and photographed using a fluorescence microscope (FV-1000; Olympus). For quantitative examination, the immunostaining results were analyzed using the Image-Pro Plus software (Version 5.1, Media Cybernetics, Inc. USA).

### Microarray analysis

To explore DEGs, high-throughput sequencing was conducted in NPCs derived from three human (NO. 006, 008, and 022). The cells were divided into TNF-α vs M1CM+ TNF-α and TNF-α vs M2CM+ TNF-α groups. The RNA library preparation and sequencing were completed by BmK Biotechnology Co., Ltd. A Bioanalyzer 2100 instrument (Agilent Technologies, USA) was used for library quality control and quantification. According to the instructions of Illumina sequencing, the 10 PM library was denatured into single stranded DNA molecules, captured on Illumina flow cells, and amplified into clusters *in situ*. Finally, 150 cycles were sequenced in PE mode on an Illumina hiseq4000 sequencer according to the manufacturer’s instructions, and double ended reads were harvested. Q30 was used for quality control, and the cut adapt software (v1.9.3) was used to remove low-quality reads. The high-quality reads obtained were compared to the human reference genome (UCSC hg19) using the hisat2 software (v2.0.4). Then, under the guidance of the GTF gene annotation file, the coffeddiff software (v2.2.1) was used. The fragments per kilobase of exon per million fragments mapped FPKM value of mRNA at the gene level were obtained as the expression profile of mRNA. Finally, the R package was used to draw a scatter chart, and the heatmap2 function of R was used for cluster analysis of DEGs with the FPKM value. The threshold set for up and down regulated genes was fold change ≥ 0.585 and FDR <0 05. The data have been uploaded to the NCBI SRA database and can be accessed to cite for these SRA data after the indicated release date: PRJNA848130

### GO functional and KEGG pathway enrichment analysis

To analyze the up and down regulated DEGs, the R package “clusterprofiler” was used to enrich the GO and KEGG pathway enrichment analysis of DEGs. The filtering threshold of functions and pathway enrichment of up- and down-regulated DEGs was set as P<0.05.

### PPI network construction and identification of hub genes

The Search Tool for the Retrieval of Interacting Genes (STRING, https://stringdb.org/) database was adopted to obtain the PPI relationships for DEGs. Briefly, a PPI network was constructed using DEGs on a string database. Then, the cytonca (version2.1.6) plug-in was used to calculate the betweenness (BC), closeness (CC), degree (DC), eigenvector (EC), lac, and network (NC) of each node in the PPI. The results of the above six indicators were sorted from large to small, and the top 30 genes were selected as hub genes.

### Statistical analysis

The values obtained are shown as the mean ± standard deviation (SD). The Mann–Whitney U test, a non-parametric test, was used to determine the statistical difference between groups. All statistical analyses were performed with the SPSS software (V11.0; SPSS, Inc., Chicago, IL, USA). Differences were considered statistically significant at P < 0.05. All quantitative results were calculated from a minimum of three biological replicates.

## Results

### Expression of M1 and M2 macrophage markers in human normal and degenerated IVD tissues

To evaluate macrophage accumulation and polarization in degenerative IVD tissues, we first performed immunohistochemical analysis to determine M1 and M2 macrophage markers, CCR7 and CD206, respectively, in human IVD tissues from control (Pfirrmann grade I Figure)) and degenerated samples (Pfirrmann grade IV, **Figure 1A**). Compared with the control tissue samples, crack or granulation IDD tissue samples showed higher CCR7 (M1-like macrophage marker)-positive cell counts (P < 0.01, **Figures 1B, C**). Furthermore, the proportion of cells positive for the M2-like macrophage marker, C206 +, was significantly elevated in degenerated IVD tissues relative to control tissues (P < 0.001, **Figures 1D, E**). These findings indicated that both M1- and M2-polarized macrophages infiltrated and accumulated in degenerated IVD tissues from patients with IDD compared with that in the normal controls.

### Effect of macrophage polarization on the proliferation ability of TNF-α-treated NPCs

**Figure 2:**
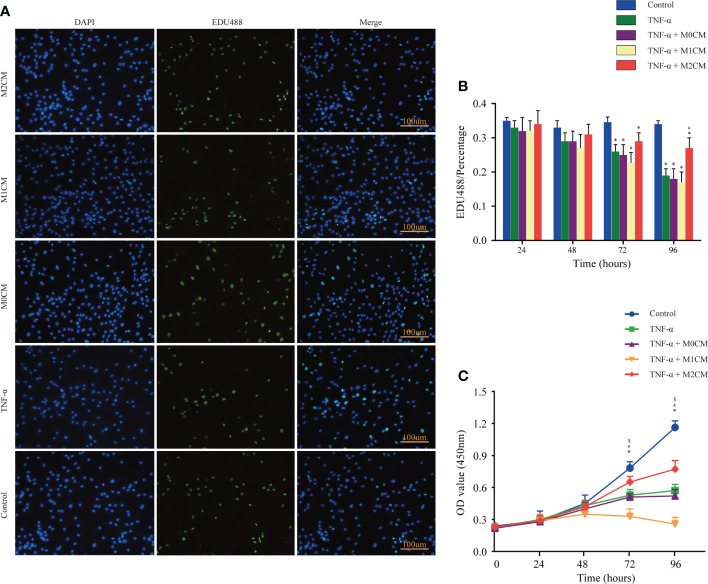
**|** Effect of macrophage polarization on the proliferation of NPCs in the TNF-α-treated environment. **(A)** Representative images showing EDU incorporation staining of NPCs in different groups. **(B)** Comparison of EDU positive staining ratios corresponding to different test compounds at different time points. **(C)** Comparison of OD values corresponding to different test compounds at different time points based on cell proliferation assays. Data are expressed as the mean ± SD (n = 4). ^*^, significant difference between the control and TNF-α-treated groups at P < 0.05; ^$^, significant difference between the TNF-α- and M1CM+ TNF-α-treated groups at P < 0.05; ^&^, significant difference between the TNF-α- and M2CM+ TNF-α-treated groups at P < 0.05. NPCs, nucleus pulposus cells; TNF-α, tumor necrosis factor-α.

To examine the role of accumulated and polarization macrophages, we analyzed NPC proliferation *via* EDU incorporation and CCK-8 assays. The results showed that the control, TNF-α-, M0CM+ TNF-α-, M1CM+ TNF-α-, and M2CM+ TNF-α- groups did not show any significant differences with respect to the percentage of EDU-positive cells and optical density (OD) values at 24 and 48 h (**Figure 2**). However, at 72 and 96 h, the TNF-α-treated groups showed decreased NPC proliferation in both test (all P < 0.05; **Figure 2**). When testing the proportion of EDU-positive cells in the TNF-α environment, the M1CM co-culture displayed an inhibitory trend, while the M2CM co-culture showed a promoting trend, with a significantly higher proportion at 96 h compared with that in the TNF-α-treated group (P < 0.05; **Figure 2B**). Similarly, following TNF-α treatment, the M2CM co-culture showed increased NPC cell proliferation, as indicated by the markedly higher OD values obtained at 72 and 96 h (both P < 0.05; **Figure 2C**), while the M1CM co-culture displayed opposite results (both P < 0.05; **Figure 2C**). These finding suggested that M1CM suppressed cell proliferation, while M2CM reversed this effect.

### Effect of macrophage polarization on ECM synthesis and pro-inflammatory mediator secretion in TNF-α-treated NPCs

**Figure 3:**
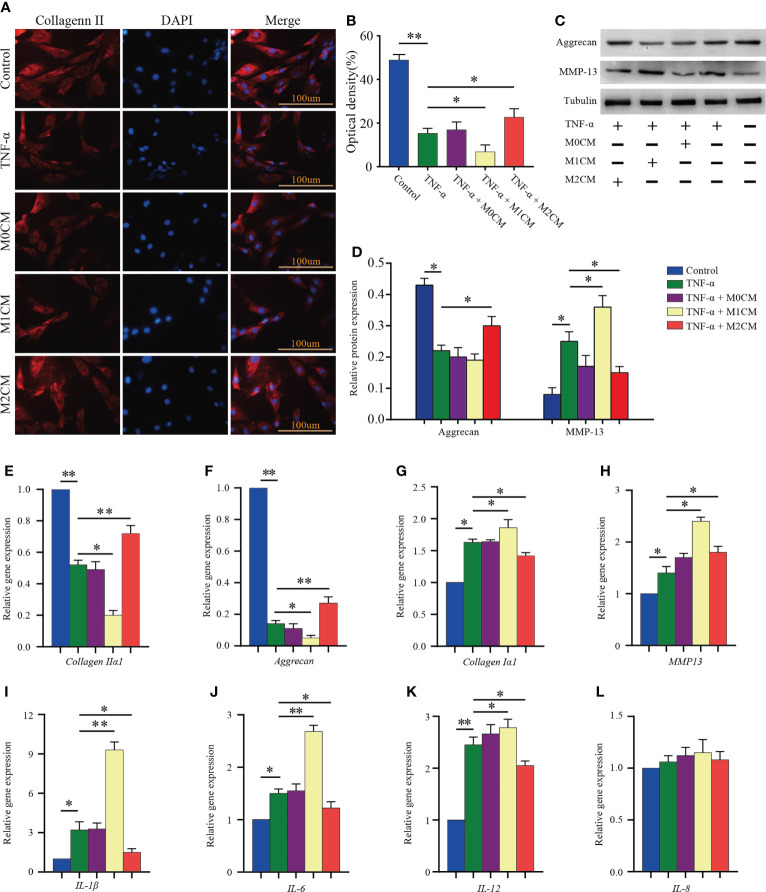
Effect of macrophage polarization on ECM synthesis and pro-inflammatory mediator secretion in TNF-α-treated NPCs. **(A)** Representative images showed collagen II immunofluorescence staining results corresponding to NPCs in different groups. **(B)** Comparison of the OD values corresponding to different test compounds after 7 days of treatment. **(C, D)** WB analysis of anabolic aggrecan and catabolish MMP-13in NPCs treated with different CMs for 7 days. **(E–L)** Gene expression of ECM components (aggrecan, collagen IIα1, and collagen Iα1), ECM-modifying enzymes (MMP-13), and pro-inflammatory mediators (IL-1β, IL-6, IL-8, and IL-12) in TNF-α-treated NPCs after 3 days based on qPCR. All data are expressed as the mean ± SD, n = 3, *P < 0.05, **P < 0.01. NPCs, nucleus pulposus cells; TNF-α, tumor necrosis factor-α; CM, condition medium; ECM, extracellular matrix; OD, optical density.

Considering ECM metabolism, it has been suggested that ECM synthesis and pro-inflammatory mediator secretion are key events associated with IDD progression (24). First, the expression of collagen II in NPCs was analyzed *via* immunofluorescence analysis (**Figure 3A**). We observed that TNF-α treatment decreased the OD value as compared to the control group (P < 0.01; **Figure 3B**). The M1CM co-culture showed an obvious decrease in OD values compared with that observed for the TNF-α-treated group (P < 0.05; **Figure 3B**), while the M2CM co-culture group showed an increase in OD values (P< 0.05; **Figure 3B**). In addition to that, the anabolism protein aggrecan showed the same trend in the TNF-α environment in western blotting analysis (P < 0.05; **Figures 3C, D**), with the M2CM co-culture slowing down this process (P < 0.05; **Figures 3C, D**). Conversely, the anabolism protein MMP-13 displayed the opposite trend, higher protein expression was observed in the M1CM group (P < 0.05; **Figures 3C, D**), and lower expression in the M2CM group (P < 0.05; **Figures 3C, D**). These findings were further validated *via* qPCR testing. The expression of ECM-related genes encoding aggrecan and collagen IIα1 was significantly downregulated in the TNF-α-treated groups relative to the control group (both P < 0.01; **Figures 3E, F**), whereas M2CM markedly upregulated their expression levels (both P < 0.01; **Figures 3E, F**). In contrast, collagen type Iα1 and MMP-13 an showed upregulated expression in the M1CM+ TNF-α-group, while the M2CM co-culture group showed a decreasing trend in this regard (all P< 0.05; **Figures 3G, H**). Lastly, the expression levels of IL-1β, IL-6, and IL12 genes were notably higher in all the TNF-α-treated groups than in the control groups (P < 0.05; P < 0.05; P < 0.01; **Figures 3I–K**). M1CM co-cultured NPCs showed significantly upregulated IL-1β, IL-6, and IL12 gene expression levels (P < 0.01; P < 0.01; P < 0.05; **Figures 3I–K**), whereas M2CM co-cultured NPCs showed significantly downregulated expression levels for all three genes compared with the TNF-α-treated NPCs (all P < 0.05; **Figures 3I–K**), except for IL-8 (**Figures 3L**). Collectively, these results revealed that M1CM treatment attenuated ECM synthesis and promoted the secretion of pro-inflammatory mediators in IDD, while in the case of M2CM treatment, reverse trends were observed.

### Effect of macrophage polarization on radiographic changes and histological scores in rat IDD models

**Figure 4:**
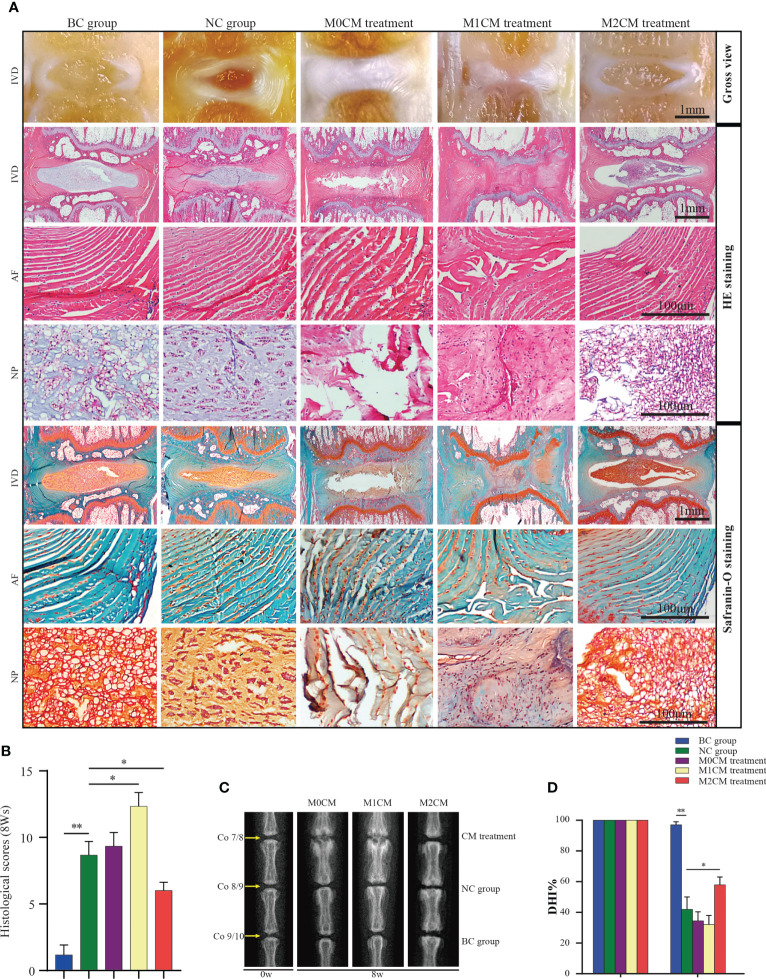
Effect of macrophage polarization on the radiographic changes and histological scores corresponding to rat coccygeal IVDs. **(A)** Representative general view, HE staining and Safranin-O/Fast Green staining images of rat coccygeal IVDs corresponding to different treatment groups (Coronal position). **(B)** Histological grading score showing changes in IVD at 8 weeks after initial puncture and different treatments. **(C)** Representative X-ray images showing rat coccygeal IVDs in different treatment groups and **(D)**, %DHI values showing changes in IVD at 8 weeks after initial puncture for different treatments. All data are expressed as the mean ± SD. n = 6, *P < 0.05, **P < 0.01. IVD, intervertebral disc; TNF-α, tumor necrosis factor-α; CM, condition medium; HE, Hematoxylin and eosin; DHI, disc height index.

To further assess the therapeutic effects of different CMs on IDD progression, rat IDD models were locally injected with CM weekly *via* needle puncture. After treatment, coronal morphology, HE staining, and Safranin-O/Fast Green staining showed the presence of well-organized NP tissues as well as an AF with clean boundaries, while NPC clusters were centralized with a layer of proteoglycan-rich matrix located at the periphery of the NP cells in BC groups (**Figures 4A, B**). After 8 weeks of needle puncture, the NC group showed a disordered morphology, with the interruption of both NP and AF tissues. Specifically, NP obviously narrowed in the disc area (**Figures 4A, B**), and compared with the NC group, M1CM injection exhibited a decreasing trend in NP tissue mass as well as AF lamella destruction, which was particularly evident at 8 weeks. However, M2CM could obviously inhibit such degeneration-related changes. The histological grading system was applied based on five categories of degenerative changes, with scores ranging from 0 points (0 in each category) for a normal disc to 15 points (3 in each category) for a severely degenerated disc as previously reported (20). Thus, we observed significantly higher histological scores for the NC group than for the BC groups at 8 weeks after the puncture operation (both P < 0.01, **Figure 4B**). Additionally, degeneration-related changes were significantly severe for the M1CM treatment groups (P < 0.01; P < 0.05, **Figure 4B** 8 weeks after puncture, whereas the scores were significantly lower for the M2CM group 8 weeks post-operation relative to the NC group (both P < 0.05, **Figure 4B**). In addition, the therapeutic potential of CM was also evaluated *via* X-ray analysis to obtain DHI% values, which are important clinical indicators of IDD (23). Compared with those for the BC group, the DHI% values corresponding to the NC puncture operation groups decreased continuously at 8 weeks (P < 0.01; **Figures 4C, D**). Furthermore, the injection of M1CM resulted in a continuous decrease in DHI%, while the M2CM injection significantly inhibited this decreasing DHI% trend throughout the experimental period relative to the NC group (P < 0.05; **Figure 4D**). These findings indicated that the rat coccygeal puncture model successfully induced IVD degeneration 8 weeks post operation. Moreover, M1CM injection promoted IDD as indicated by the histological grading scores and DHI% analyses, whereas the M2CM treatment showed a contrasting effect throughout the treatment period.

### Effect of macrophage polarization on ECM metabolism and pro-inflammatory mediator secretion in rat IDD models

**Figure 5:**
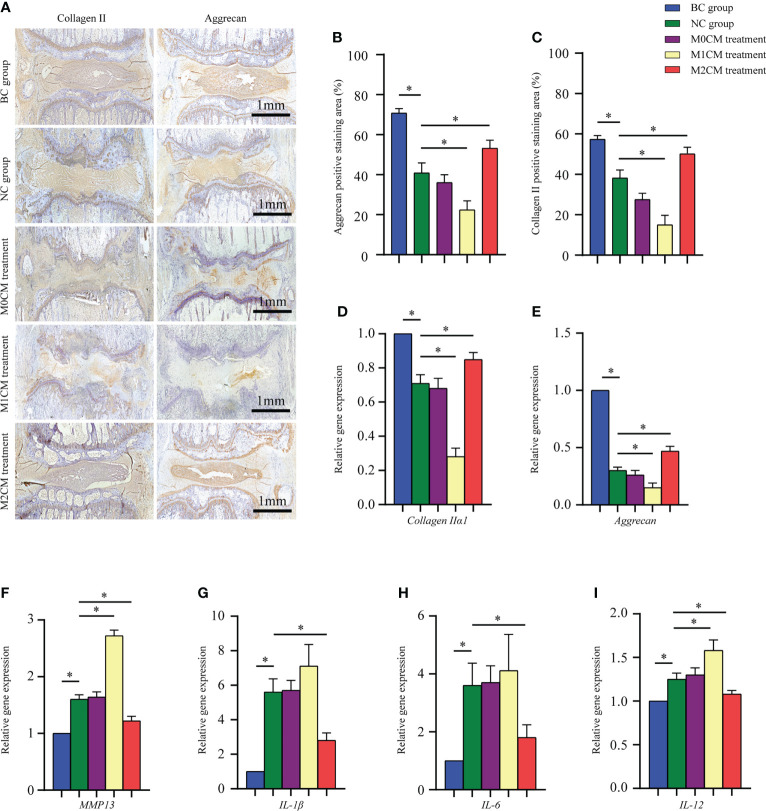
Effect of macrophage polarization on ECM metabolism and pro-inflammatory mediator secretion in rat coccygeal IVDs. **(A)** Representative immunohistochemical images showing rat coccygeal IVDs in different groups. **(B, C)** Semi-quantification analysis of collagen II and aggrecan staining in rat coccygeal IVDs 8 weeks after initial puncture and different treatments. **(D–I)** Effect of macrophage polarization on the gene expression of ECM components (aggrecan and collagen IIα1), ECM-modifying enzymes (MMP-13), and pro-inflammatory mediators (IL-1 and IL-12) in rat coccygeal IVDs 8 weeks after puncture operation and different treatments. All data are expressed as the mean ± SD, n = 6, *P < 0.05, **P < 0.01. IVD, intervertebral disc; ECM, extracellular matrix; TNF-α, tumor necrosis factor-α; CM, condition medium.

To evaluate the effect of macrophage polarization on the disc matrix, IVD sections were stained with antibodies against collagen II and aggrecan. As shown in **Figure 5A**, immunohistochemical staining showed that AF and NP corresponding to the BC groups were strongly positive for collagen II and aggrecan; however, the staining intensity decreased significantly for the NC group 8 weeks after puncture (both P < 0.05, **Figures 5B, C**). Additionally, our results indicated that 8 weeks after initial puncture, M1CM downregulated both collagen II and aggrecan expression (both P < 0.05, **Figures 5B, C**); however, this effect was inhibited by the M2CM treatment (both P < 0.05, **Figures 5B, C**). Consistent with this observation, gene expression analysis exhibited similar results for both collagen IIα1 and aggrecan genes (All P < 0.05, **Figures 5D, E**). We also investigated the expression of ECM catabolic and pro-inflammatory mediator-related genes 8 weeks after puncture. The results thus obtained showed that *MMP13* was significantly upregulated in the NC group relative to the BC group 8 weeks after puncture (P < 0.05, **Figure 5F**). The M1CM group showed increased MMP13 mRNA levels, with markedly higher expression levels (P < 0.05, **Figure 5F**), while the M2CM group showed significant MMP13 mRNA expression downregulation relative to the NC group (P < 0.05, **Figure 5F**). Furthermore, the M1CM group showed increased expression levels for the pro-inflammatory mediators, IL-1, IL-6, and IL-12 relative to the NC group, with a markedly higher IL-12 expression level (P < 0.05, **Figures 5G–I**). Conversely, the M2CM group showed significantly lower expression levels for these three mRNAs than the NC group (all P < 0.05, **Figures 5G–I**). Taken together, these findings suggested that M1CM exerted destructive effects in IDD, while M2CM could alleviate them.

### Identification of DEGs

**Figure 6:**
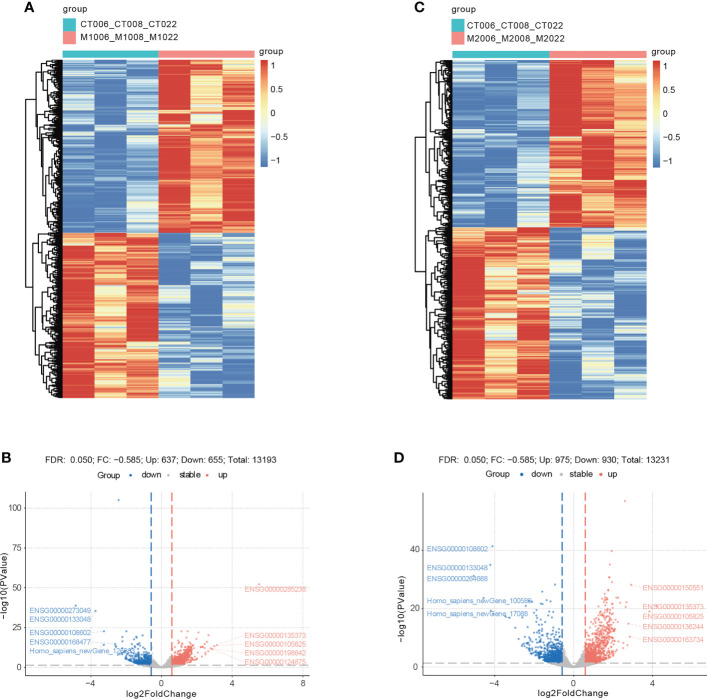
Cluster analysis and volcano plot showing differentially expressed genes (DEGs) in M1CM or M2CM treated NPCs. **(A, B)** Cluster analysis heat map of DEGs, where each small square represents each mRNA, and its color represents the amount of mRNA expression. The greater the amount of mRNA expression, the darker is the color (red indicates high expression, blue indicates low expression). The first line indicates sample grouping, blue indicates treatment samples, red indicates control samples. Each row represents the expression of each mRNA in different samples, and each column represents the expression of all differential mRNAs in each sample. The tree on the left shows the cluster analysis results of different mRNAs from different samples. **(C, D)** Volcano map of DEGs, where each dot represents an mRNA. A red dot indicates that the gene expression is upregulated, a blue dot indicates that the mRNA expression is down regulated, and a gray dot indicates that there is no significant difference in the mRNA levels.

When comparing the M1CM+ TNF-α group with the TNF-α group, a total of 1292 DEGs were detected, including 637 upregulated genes and 655 downregulated genes (**Figures 6A, B**). The DEGs refer to those genes that exhibited increased or decreased expression in M1CM co-cultured NPCs. The mostest upregulated gene was ETS-homologous factor (EHF) (fold change = 3.14), followed by TFPI2, and CXCL6. The most downregulated gene was CHI3L1(fold change = −3.74), followed by Homo_ sapiens_ newGene_126363, and ALDH3A1. The top 10 up- and downregulated genes are shown in **Table 1**. When comparing the M2CM+ TNF-α group with TNF-α group, a total of 1905 DEGs were detected, including 975 upregulated genes and 930 downregulated genes (**Figures 6C, D**). The DEGs refer to those genes that exhibited increased or decreased expression in M2CM co-cultured NPCs in the TNF-α environment. The most upregulated gene was EHF (fold change = 4.2), followed by TFPI2, and LYPD1. The most downregulated gene was Homo_sapiens_newGene_100589 (fold change = −4.57), followed by CHI3L1, and Homo_sapiens_newGene_17088. The top 10 up- and downregulated genes are shown in **Table 2**.

**Table 1:** The top 10 upregulated and downregulated genes in M1CM treated NPCs.

Gene names	Fold change	P value
Upregulated genes		
EHF	3.14	9.82E-15
TFPI2	2.98	5.14E-14
CXCL6	2.87	3.37E-12
KLHL9	2.78	1.23E-12
MMP3	2.66	6.27E-21
CXCL3	2.64	7.71E-11
PDE7B	2.57	8.89E-17
SYS1-DBNDD2	2.49	7.56E-09
CXCL1	2.48	5.09E-10
IL6	2.44	7.04E-13
Downregulated genes		
CHI3L1	-3.74	3.75E-36
Homo_sapiens_newGene_126363	-3.27	6.54E-15
ALDH3A1	-3.26	2.12E-23
TNXB	-2.63	2.24E-15
CLEC3B	-2.61	2.60E-14
ITIH5	-2.57	1.34E-13
Homo_sapiens_newGene_108633	-2.47	1.13E-08
DUSP3	-2.43	9.16 E-06
Homo_sapiens_newGene_10962	-2.36	3.78E-09
ABCA10	-2.25	6.66E-10

Filter criteria for significant difference gene: |fold change| >0.585, P value < 0.05.

**Table 2:** The top 10 upregulated and downregulated genes in M2CM treated NPCs.

Gene names	Fold change	P value
Upregulated genes		
EHF	4.2	1.16E-21
TFPI2	3.84	9.45E-20
LYPD1	2.91	6.98E-29
CXCL3	2.85	4.17E-11
IL6	2.78	1.37E-15
CXCL6	2.73	3.54E-08
PDE7B	2.68	6.32E-23
IFI6	2.64	2.37E-21
TMEM158	2.6	1.20E-57
CXCL1	2.52	5.66E-09
Downregulated genes		
Homo_sapiens_newGene_100589	-4.57	2.07E-24
CHI3L1	-4.23	9.10E-36
Homo_sapiens_newGene_17088	-4.21	5.27E-20
ALDH3A1	-4.12	3.67E-42
KCNB1	-4.09	5.84E-19
Homo_sapiens_newGene_123586	-3.47	4.22E-18
ITIH5	-3.25	1.39E-17
CLEC3B	-2.96	3.49E-14
Homo_sapiens_newGene_35847	-2.79	2.40E-10
SLC47A2	-2.63	6.24E-08

Filter criteria for significant difference gene: |fold change| >0.585, P value < 0.05.

### GO functional and KEGG pathway enrichment analysis

**Figure 7:**
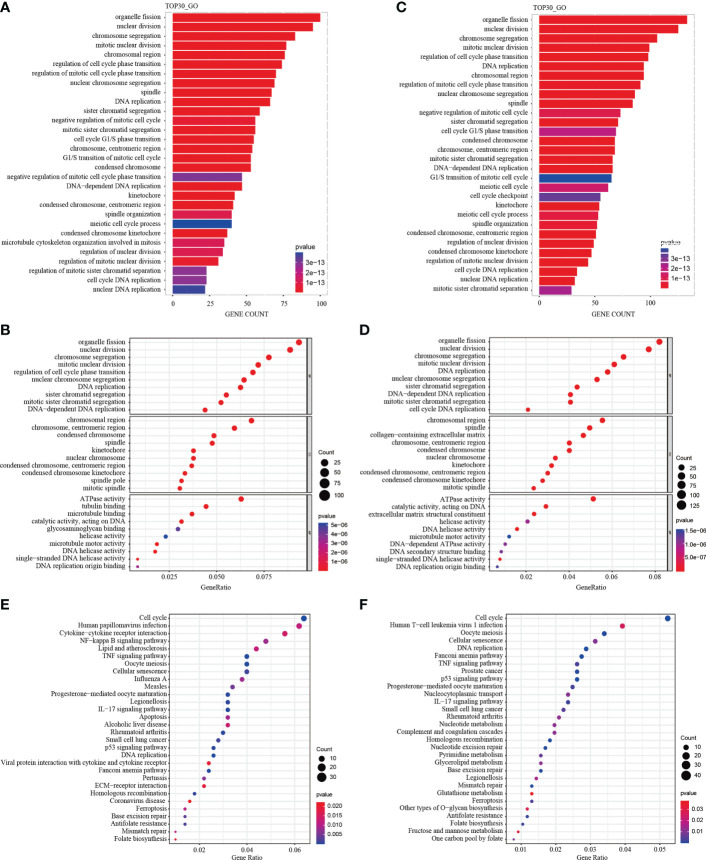
GO functional and KEGG pathway enrichment analysis of differentially expressed genes (DEGs) in M1CM or M2CM treated NPCs. The GO terms and KEGG pathway names are shown on the y-axis. Top 30 most significantly enriched GO terms of NPCs treated with M1CM **(A)** or M2CM **(C)** are displayed in a GO functional enrichment strip map. The length of the bars on the x-axis represents the gene counts, and the GO terms are shown on the y-axis. The bubble chart of the top 10 most significantly enriched GO terms of NPCs treated with M1CM **(B)** or M2CM **(D)** are displayed in terms of BPs, MF and CCs respectively. The x-axis represents the gene proportion in the annotation pathway, and the y-axis represents the GO terms. The color is determined by the P value, and the size is determined by the number of genes in the annotation pathway. **(E, F)** KEGG function enrichment bubble chart. The x-axis represents the ratio of genes in the annotation pathway, and the y-axis represents the KEGG pathway, the color is determined by the P value, and the size is determined by the number of genes in the annotation pathway.

A total of 1292 differential genes were enriched for GO function analysis in M1CM treated NPCs. The top 30 pathways were selected according to P value from low to high, and arranged by gene counts (**Figure 7A**). The GO analysis showed that, the DEGs were mainly enriched in response to organelle fission, nuclear division and chromosome segregation in terms of biological processes (BPs) (**Figure 7B**). In terms of cellular components (CCs), DEGs were mainly enriched in the chromosomal region, chromosome centromeric region, and condensed chromosome (**Figure 7B**). In terms of molecular function (MF), DEGs were mainly enriched in ATPase, tubulin binding and microtubule (**Figure 7B**). The KEGG pathway analysis showed that the DEGs were enriched in 44 pathways; the most significant pathway was the cell cycle pathway, and the top 30 significant pathways are shown in **Figure 7E**. In M2-CM treated NPCs, a total of 1905 differential genes were enriched for GO function, and the top 30 channels were selected (**Figure 7C**). The results showed that the DEGs were mainly enriched in response to organelle fission, nuclear division, and chromosome segregation in terms of BP (**Figure 7D**), In terms of CC, DEGs were mainly enriched in chromosomal region, spindle and collagen-containing extracellular matrix (**Figure 7D**). In terms of MF, DEGs were mainly enriched in ATPase activity, catalytic activity acting on DNA and extracellular matrix structural constituent (**Figure 7D**). The KEGG pathway analysis showed that DEGs were enriched in 36 pathways; among them, the most significant pathway was the cell cycle pathway, and the top 30 significant pathways are shown in **Figure 7F**.

### PPI analysis and hub gene screening

**Figure 8:**
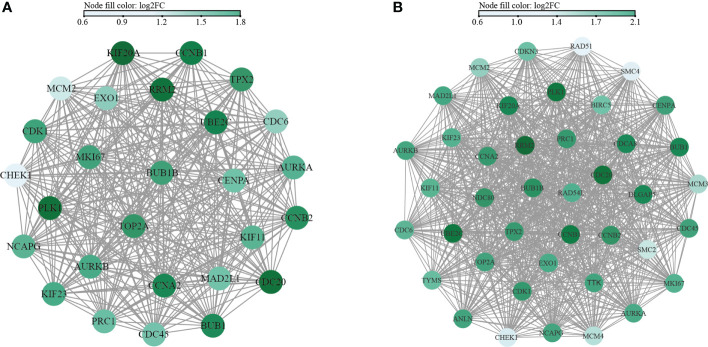
Network interaction diagram of hub genes in M1CM **(A)** and M2CM **(B)** groups. The nodes represent the hub genes, and the edges represent the protein protein interaction. The color is determined by the results of the six indicators from large to small, and the greater the score, the darker is the color.

Functional PPI analysis is essential to interpret the molecular mechanisms of key cellular activities. After running the CytoHubba plug-in, the hub genes were identified by six calculation methods. A total of 27 hub genes were obtained in the M1CM group (**Figure 8A**). Similarly, a total of 41 hub genes were obtained in the M2CM group (**Figure 8B**). The five most significant genes in the M1CM group were PLK1, KIF20A, RRM2, CDC20, UBE2C and those in the M2CM groups were RRM2, CCNB1, CDC20, PLK1, and UBE2C.

## Discussion

Macrophages were detected to infiltrate, polarize, and secrete cytokines that act additively or synergistically to regulate NPCs in IDD process (4). Hence, a promising therapeutic strategy would be to regulate this process by blocking the actions of all, or at least multiple cytokines. Compared with antagonistic or neutralizing inflammatory factors, the cell co-culture model offers the possibility to clarify the full spectrum of the molecular changes caused by macrophages, which is closer with IDD environment (25, 26). We used a spectrum of macrophages, including M0, M1, and M2 cells, by analyzing cell proliferation, ECM synthesis, and the secretion of pro-inflammatory factors, as well as exploring the potential mechanism in the IDD process. To the best of our knowledge, this is the first study in which a comprehensive analysis of macrophage subtypes in IDD was conducted. Our results indicated that macrophages exert biological effects on IDD primarily in their polarized states rather than as macrophages themselves. Specifically, M1CM inhibited NPC proliferation and exacerbated IVD degeneration in experimental IDD models, while M2CM attenuated IDD development. In this process, a total of 1038 DEGs were identified in M1CM treated NPCs, and 1905 DEGs in the M2CM group. Of these, cell cycle was the most significant pathway enriched in KEGG enrichment, and organelle fission, nuclear division, and chromosome segregation were highly enriched in GO functional enrichment in both treatment NPCs. Finally, there were 27 hub genes in the M1CM group and 41 hub genes in the M2CM, with top six hub genes were PLK1, CDC20, KIF20A, UBE2C, RRM2 and CCNB1. Our results may facilitate the further clarification of the biological effects of macrophage polarization, explore potential mechanism underlying IDD, and provide novel insights into the effective therapeutic potential.

In this study, we mimicked the inflammatory environment *via* TNF-α treatment *in vitro* and performed a caudal IVD acupuncture model *in vivo*, both of which have been widely used in the study of IDD (27, 28). To eliminate the effects of differences arising from factors, such as weight, infection status, activity level, and immune competence among rats, we obtained control and experimental tissue segments from the same animal. We observed that TNF-α-induced IDD; further, the rat coccygeal IDD model showed degenerated phenotypes and pro-inflammatory cytokine secretion, suggesting that the *in vitro* and *in vivo* models used in this study were successful and reliable.

Macrophages regulated growth factors, pain-related molecules, and IVD markers (13, 14). However, previous studies often tested in the inflammatory environment, which probably induce macrophage polarization and make the results ambiguous in some extend. A recent study by Li et al. revealed M1 macrophage polarization induced IDD (29), and M2a macrophages promote ECM metabolic imbalances in rat IDD (30). Nonetheless, the spectrums of macrophages that mimic actual environment of IDD are lacking. In this study, the M1 and M2 macrophage polarization markers CCR7 and CD206 were higher expression in human degenerated NP tissues, and M1CM exacerbate IDD by inhibiting cell proliferation, cell anabolism, and promoting pro-inflammatory mediator secretion, while M2CM showed protection effects by suppressing TNF-α-induced ECM degradation and inflammation reaction. These findings suggested that inhibiting pro-inflammatory M1 macrophages or enhancing the performance of anti-inflammatory M2 macrophages, rather than the macrophage themselves is relevant targets to consider, when developing therapies for inhibiting IDD.

Reports regarding specific molecules or the associated mechanisms of macrophage in IDD are limited (31). In the present study, high throughput sequencing was used to identify DEGs between control and CMs treated NPCs in the TNF-α environment. Among the down-regulated genes, EHF was the most significantly altered gene in both groups, while among the up-regulated genes, CHI3L1 in M1CM treated NPCs and Homo_sapiens_newGene_100589 showed the most significantly changes in M1CM and M2CM treated NPCs respectively. EHF is known to play key roles in cell proliferation and cell differentiation, but the role in IDD is unclear. CHI3L1 is strongly expressed by macrophages and mediate pro-inflammatory effects in many inflammatory diseases. M2a macrophages secreted CHI3L1 proteins to promote ECM metabolic imbalance in NP cells (30). Our results found M1CM treating NPCs also high expression CHI3L1, highlighting its important role in IDD. Homo_sapiens_newGene_100589 was firstly found highest expressed in M2CM groups, but its role need further illuminated. We also detected GO terms enrichment in several terms such as organelle fission, nuclear division and chromosome segregation in both groups, and the most significant pathway in KEGG pathway analysis is cell cycle. Aside from that, collagen-containing extracellular matrix and catalytic activity acting on DNA and extracellular matrix structural constituent were highly enriched in M2CM group. This is according with our results that M2CM slowed down IDD by suppressing TNF-α-induced ECM degradation, indicating potential in promoting ECM synthesis. Taken together, cell cycle and cell proliferation were highly enriched in GO functional and KEGG pathway enrichment, indicating us the most potential targets in treating IDD.

The hub genes were analyzed in the PPI network by six calculation methods. The top five derived hub genes were PLK1, KIF20A, RRM2, CDC20, and UBE2C in M1CM groups and RRM2, CDC20, CCNB1, PLK1, and UBE2C in M2CM groups. Polo like kinase 1 (PLK 1) plays an important role in the regulation of the cytoskeleton network during mitosis (32), and li et al. demonstrated PLK1 expression was decreased in NPCs of degenerative IVDs (33). In addition, CCNB1 is a member of cyclin family, which is responsible for cell proliferation and differentiation (34). Its elimination inhibits the proliferation of IVD cells, revealing that CCNB1 is an important regulator of the G2/M phase of NP cells (35). Finally, Kinesin-like family member 20A (KIF20A), is considered as a significant molecule for cell cycle regulation (36). Zhao et al. demonstrated that KIF20A can promote tumor cell proliferation and inhibit apoptosis *in vivo* and *in vitro* (37). Likewise, Zhang et al. [60] confirmed that the mRNA levels of KIF20A were upregulated in degenerative NP tissue than in healthy NP tissue (38). These findings indicate that KIF20A may be a novel target associated with NP cell degeneration. Interestingly, Ubiquitin-conjugating enzyme e2c (UBE2C), Ribonucleoside-diphosphate reductase subunit M2 (RRM2), and Cell division cycle 20 homologue (CDC20), holding important role in the regulation of cell cycle process (39–41), were firstly found in IDD and needed further study. In conclusion, we used bioinformatics analysis to identify PLK1, CDC20, KIF20A, UBE2C, RRM2, and CCNB1 as hub genes related to IDD, thereby providing new insights in treating IDD.

**Figure 9:**
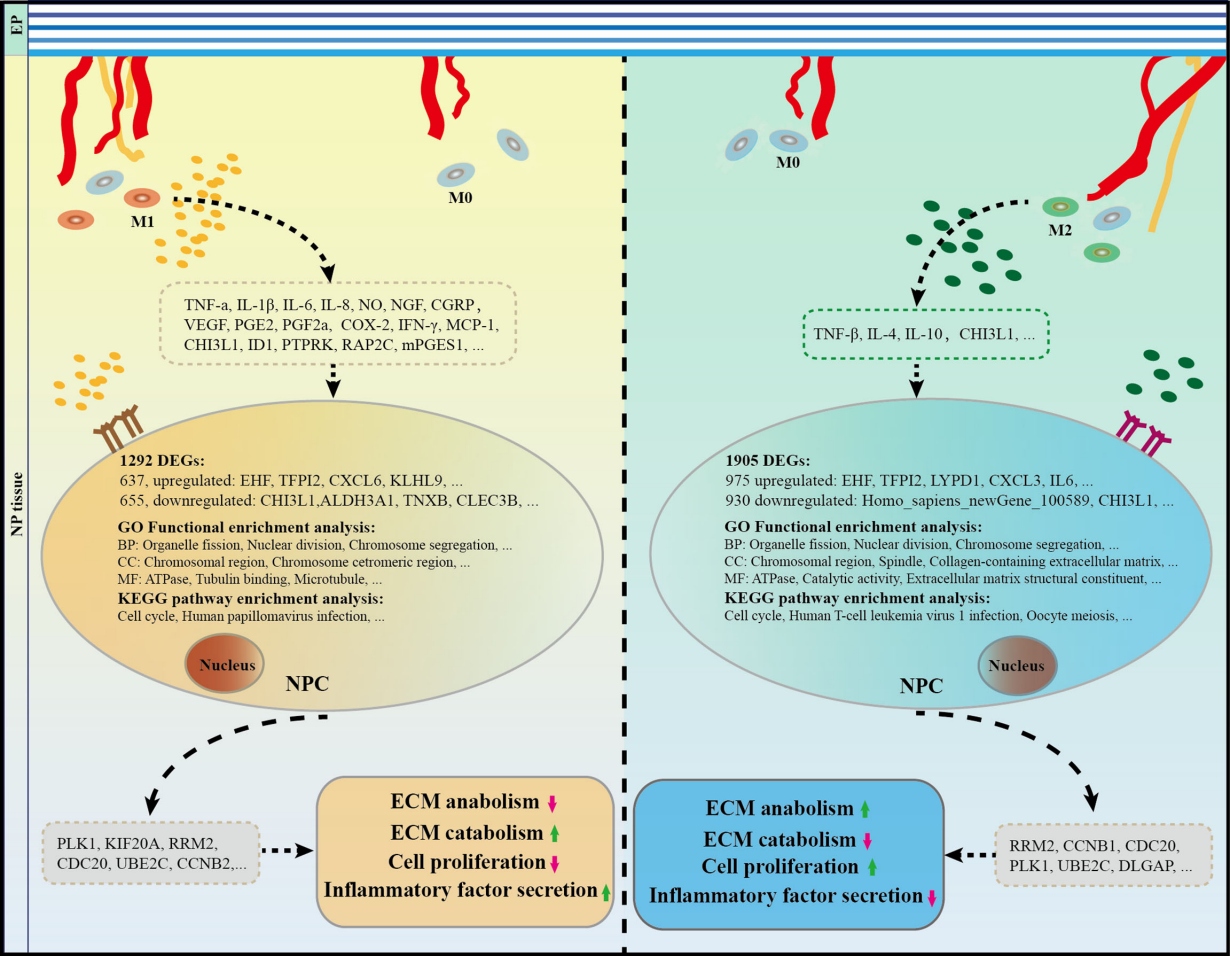
The role and potential mechanism of M1 and M2 macrophages in the process of IDD. Macrophages take part in IDD mainly in M1 and M2 polarization rather than macrophage themselves. Specifically, M1CM inhibits cell proliferation and exacerbates IVD degeneration, while M2CM promote cell proliferation and attenuates IDD development. A total of 1038 DEGs were identified in M1CM treated NPCs, and 1905 DEGs in the M2CM group. Cell cycle was the most significant pathway enriched in KEGG enrichment, and organelle fission, nuclear division, and chromosome segregation were highly enriched in GO functional enrichment in both treatment NPCs. Finally, the five most significant genes in the M1CM group were PLK1, KIF20A, RRM2, CDC20, UBE2C and those in the M2CM groups were RRM2, CCNB1, CDC20, PLK1, and UBE2C. IDD, intervertebral disc degeneration; IVD, intervertebral disc.

Lastly, a range of cytokines related to M1 or M2 polarized macrophages in IVDs has been identified based on a review of related literature (**Table 3**). Specifically, high expression levels of M1-related cytokines, such as TNF-α, TNFR1, IL-6, IL-8, NGF, VEGF, COX-2, MCP-1, PGE2, NLRP3, and IFN-γ, promote IDD. Conversely, M2 macrophages may secrete cytokines, such as IL-4, IL-10, and TGF-β, which inhibit the IDD process. Furthermore, it has been reported that if properly balanced, both of M1 and M2 macrophages may contribute to IDD repair/regeneration as has already been demonstrated for other tissues (16). Taken all into consideration, it can be concluded that with IVD tissue degeneration, there were higher macrophage infiltration, polarization, and generating a special types of microenvironment which interact with NPCs through cytokines. This process brought abnormal gene expression of PLK1, CDC20, KIF20A, UBE2C, RRM2, and CCNB1 and pathway of cell cycle, and amplification of multiple down-stream cascades from pro-inflammatory M1 macrophages, together with the cytokines they secrete, aggravate IDD pathology, hinder recovery, and cause pain, while anti-inflammatory and remodeling M2 macrophages show an increasing expression of growth factors and anti-inflammatory agents (**Figure 9**).

**Table 3:** Summary of the main biological effects and the main components of macrophages in IDD process.

Cell sources	Species	Biological effects	Main components	
***In vivo***				
F4/80+ CD11b+ cells (44)	Mice	NGF expression was increased in both inflammatory and non-inflammatory states following IVD injury.	NGF	
F4/80+ CD11b+ cells (45)	Mice	Macrophages in the injured IVDs produced inflammatory cytokines, but not growth factors. Macrophage-derived inflammatory cytokines regulate growth factors and pain-related molecules.	NGF, VEGF, COX-2, and mPGES1	
F4/80+ cells (26)	Mice	Puncture injury induced an acute increase in dorsal macrophage infiltration that persisted for 12 months. Increased nerve fiber marker (CGRP) density was observed primarily on the dorsal aspect of bulging and herniated IVDs and increased with DD severity.	CGRP	
CD86+ CD11b+ F4/80+ cells (46)	Mice	M1 macrophages following IVD injury originate from recruited macrophages, and resident macrophages may produce MCP-1 to induce M1 macrophages.	MCP-1	
CD14+ cells (47)	Human	CD14 (+) cells directly and indirectly contributed to inflammatory cytokine and pain related molecule expression in human degenerated IVD.	TNF-a, IL-1β, IL-6, NGF, and CGRP	
***In vitro***				
Co-culture THP-1 cells and Human AF cells (48)	Human	Co-culture with macrophages up-regulated AF cell secretion of IL-8 dose-dependently and down-regulated NO to TNF-α or IL-1 β stimulation.	IL-6, IL-8, NO	
Co-culture THP-1 cells and human AF cells (49)	Human	Annular injury can result in macrophage infiltration, and this can cause enhanced inflammatory mediator and VEGF production by AF cells. The p38 MAPK pathway signals are responsible for much of IL-6 and PG secretion from AF cells.	VEGF, IL-6, PGE2, PGF2a	
Co-culture of macrophages RAW 264.7 and rat AF or NP cells (14)	Rat	The biologic interactions between infiltrating macrophages and native disc cells under degenerated disc inflammatory environment lead to an increasingly severe inflammatory conditions.	IFN-γ, IL-1β, TNF-a	
Co-culture of macrophages and rat IVD culture model (50)	Rat	Both non-degenerated discs and discs with several degrees of degeneration were able to produce dramatically high amounts of PGE2 and IL-6 when they interacted with macrophage.	PGE2, IL-6	
Co-culture of human macrophages and bovine IVD organ culture model (13)	Bovine	Macrophages seem to exhibit a more pro-inflammatory profile, producing more IL-6.	IL-6	
Co-culture of M1 macrophages and human NP cells with Magnoflorine (29)	Human	Magnoflorine alleviates M1 macrophage-mediated NP cells damage by inactivating the HMGB1-MyD88-NF-κB pathway and NLRP3 inflammasome.	NLRP3, IL-1β, IL-6, TNF-α, IL-18	
Co-culture of M2a macrophages and NP cells. (30)	Human	M2a macrophages promoted the expression of catabolism genes and suppressed the expression of anabolism genes in NP cells through CHI3L1.	CHI3L1	
Bioinformatic analysis of macrophage infiltration in LDH (51)	Human	Compared with control tissue, LDH tissue contained a higher proportion of macrophages.	ID1, PTPRK, and RAP2C	

NGF: nerve growth factor; VEGF: vascular endothelial growth factor; NLRP3: NOD-like receptor 3; PGE2: prostaglandin (PG)E2; COX-2: cyclooxygenase; mPGES1: microsomal prostaglandin E synthase-1; MCP-1: Monocyte chemoattractant protein-1; CGRP: calcitonin gene-related peptide; ID1: inhibitor of DNA binding 1; CHI3L1: chitinase 3-like 1; TNF-α: tumor necrosis factor (TNF) -alpha, IL-1β: interleukin (IL) -1beta.

This study has several limitations. First, even though TNF-α can mimic the inflammatory effects of IDD to some extent, the actual environment associated with this condition is complex and involves several factors that are difficult to completely recapitulate. Second, the validation study was performed using an AF-punctured animal model, which has characteristics that are different from those associated with the complex pathological process of human disc degeneration. Presently, animal models are generally established *via* mechanical interventions. Alternatively, spontaneous degeneration models are used (42). Third, the use of rat models does not reflect nutritional deficiencies, collagen disorganization, or cell death, which is often seen in chronic human IDD (43). In addition, the rat tail is non-weight, which differs from that of the human lumbar spine. Finally, *in vivo* and *in vitro* experiments are required to further validate our findings, which will be the focus of future research.

## Data availability statement

The original contributions presented in the study are included in the article/**Supplementary Material**. Further inquiries can be directed to the corresponding author.

## Ethics statement

Written informed consent was obtained from the individuals for the publication of any potentially identifiable images or data included in this article.

## Author contributions

X-CL, M-SW, and C-MH conceived and designed the experiments. X-CL, S-JL, T-LZ, D-QT, R-XT, Q-ZX, JL, and WF collected, analyzed, and interpreted the data, and wrote the manuscript. C-MH and M-SW provided reagents and reviewed the manuscript for intellectual content. All authors contributed to the article and approved the submitted version.

## Funding

This work was supported by the National Natural Science Foundation of China (Grant No 81802130), China Postdoctoral Science Foundation (2018M630968), and Natural Science Foundation of Guangdong Province (2018A030310462).

## Acknowledgments

The authors would like to thank Jin-Mei Liao from the Department of Orthopedics, Gaozhou People’s Hospital, for their invaluable assistance in obtaining human NP specimens.

## Conflict of interest

The authors declare that the research was conducted in the absence of any commercial or financial relationships that could be construed as a potential conflict of interest.

## Publisher’s note

All claims expressed in this article are solely those of the authors and do not necessarily represent those of their affiliated organizations, or those of the publisher, the editors and the reviewers. Any product that may be evaluated in this article, or claim that may be made by its manufacturer, is not guaranteed or endorsed by the publisher.
