# Supplementary material for: Macrophage polarization regulates intervertebral disc degeneration by modulating cell proliferation, inflammation mediator secretion, and extracellular matrix metabolism
Source: Front Immunol. 2022 Aug 18;13:922173. doi: 10.3389/fimmu.2022.922173 (PMC9433570; doi:10.3389/fimmu.2022.922173)
Supplement: Supplementary file 1 [file Table_1.docx]

| **Table S1: Detailed characteristics of the enrolled patients** | | | | | |
| --- | --- | --- | --- | --- | --- |
| Case No. | Gender | Age(years) | Diagnosis | Disc level | Pfirrmann grade |
| Case 1 | Female | 40 | VBT | L4-L5 | I |
| Case 2 | Female | 12 | AIS | T12-L1 | I |
| Case 3 | Male | 49 | VBT | L5-S1 | I |
| Case 4 | Male | 5 | CS | L4-L5 | I |
| Case 5 | Female | 12 | AIS | L1-L2 | I |
| Case 6 | Female | 46 | LDH | L3-L4 | III |
| Case 7 | Male | 27 | LDH | L4-L5 | III |
| Case 8 | Male | 58 | LDH | L5-S1 | IV |
| Case 9 | Female | 49 | LDH | L4-L5 | IV |
| Case 10 | Female | 51 | LDH | L4-L5 | IV |
| Case 11 | Male | 60 | LDH | L5-S1 | IV |
| Case 12 | Male | 62 | LDH | L5-S1 | IV |
| Case 13 | Female | 65 | LDH | L4-L5 | V |
| LDH, lumbar disk herniation. VBT: Vertebral body tumor; AIS: Adolescent idiopathic scoliosis; CS: Congenital scoliosis | | | | | |
