# Supplementary material for: Macrophage polarization regulates intervertebral disc degeneration by modulating cell proliferation, inflammation mediator secretion, and extracellular matrix metabolism
Source: Front Immunol. 2022 Aug 18;13:922173. doi: 10.3389/fimmu.2022.922173 (PMC9433570; doi:10.3389/fimmu.2022.922173)
Supplement: Supplementary file 2 [file Table_2.docx]

| **Table S2: Detailed characteristics of the enrolled patients** | | | | | |
| --- | --- | --- | --- | --- | --- |
| Case No. | Gender | Age(years) | Diagnosis | Disc level | Pfirrmann grade |
| Case 1 | Male | 20 | LDH | L4-L5 | III |
| Case 2 | Male | 26 | LDH | L4-L5 | III |
| Case 3 | Male | 39 | LDH | L5-S1 | III |
| Case 4 | Male | 27 | LDH | L4-L5 | III |
| Case 5 | Male | 31 | LDH | L4-L5 | III |
| Case 6 | Male | 30 | LDH | L5-S1 | III |
| Case 7 | Male | 42 | LDH | L5-S1 | III |
| Case 8 | Male | 25 | LDH | L4-L5 | III |
| LDH, lumbar disk herniation. | | | | | |
