# Supplementary material for: Macrophage polarization regulates intervertebral disc degeneration by modulating cell proliferation, inflammation mediator secretion, and extracellular matrix metabolism
Source: Front Immunol. 2022 Aug 18;13:922173. doi: 10.3389/fimmu.2022.922173 (PMC9433570; doi:10.3389/fimmu.2022.922173)
Supplement: Supplementary file 3 [file Table_3.docx]

| **Table S3 Primers for target genes** | | | |
| --- | --- | --- | --- |
| Gene | Species | Forward sequence | Reverse sequence |
| Aggrecan | Human | 5′-GTCAGATACCCCATCCACACTC-3′ | 5′-CATAAAAGACCTCACCCTCCAT-3′ |
| Collagen IIα1 | Human | 5′-GGTAAGTGGGGCAAGACTGTTA-3′ | 5′-TGTTGTTTCTGGGTTCAGGTTT-3′ |
| Collagen Iα1 | Human | 5′-CCTGGAAAGAATGGAGATGATG-3′ | 5′-ATCCAAACCACTGAAACCTCTG-3′ |
| MMP-13 | Human | 5′-TGGAAGGATGCCTTTTTTTCTC-3′ | 5′-CACCCTCCCCAAGTATCAATAGG-3′ |
| IL-1β | Human | 5′-CTTATTACAGTGGCAATGAGGATG-3′ | 5′-CTTTCAACACGCAGGACAGGTACA-3′ |
| IL-6 | Human | 5′-ATGCCTGACCTCAACTCCACT-3′ | 5′-GCCACCCAGCTGCAAGATTTC-3′ |
| IL-8 | Human | 5′-TTTTGCCAAGGAGTGCTAAAGA-3′ | 5′-AACCCTCTGCACCCAGTTTTC-3′ |
| IL-12 | Human | 5′-GCAGCTTCTTCATCAGGGAC-3′ | 5′-AGGGTACTCCCAGCTGACCT-3′ |
| GAPDH | Human | 5′-AGAAGGCTGGGGCTCATTTG-3′ | 5′-AGGGGCCATCCACAGTCTTC-3′ |
| β-actin | Human | 5′-GTGGGGCGCCCCAGGCACCA-3′ | 5′-CTTCCTTAATGTCACGCACGATTTC-3′ |
| 18S | Human | 5′-GCCGCTAGAGGTGAAATTCTTG-3′ | 5′-CATT CTTGGCAAATGCTTTCG-3′ |
| Aggrecan | Rat | 5′-CCCTACCCTTGCTTCTCCA-3′ | 5′-CTTGAGAGGCACTCATCAATGT-3′ |
| Collagen IIα1 | Rat | 5′-GGCTCCCAGAACATCACCTA-3′ | 5′-GCCCTCATCTCCACATCATT-3′ |
| MMP-13 | Rat | 5′-AGGCCTTCAGAAAAGCCTTC-3′ | 5′-GAGCTGCTTGTCCAGGTTTC-3′ |
| IL-1β | Rat | 5′-GGGTTGAATCTATACCTGTCCTGTGT-3′ | 5′-TTGGGTATTGTTTGGGATCCA-3′ |
| IL-6 | Rat | 5′-AAGCAGGTCCAGCCACAATGTAG-3′ | 5′-CCAACTGACTTTGAGCCAACGAG-3′ |
| IL-12 | Rat | 5′-ACCCTCACCTGTGACAGTCC-3′ | 5′-GACAGAGATGCTCGTCCACA-3′ |
| GAPDH | Rat | 5′-TGCCACTCAGAAGACTGTGG-3′ | 5′-TTCAGCTCTGGGATGACCTT-3′ |
| β-actin | Rat | 5′-GGAGATTACTGCCCTGGCTCCTA -3′ | 5′-GACTCATCGTACTCCTGCTTGCTG -3′ |
| 18S | Rat | 5′-AAGTTTCAGCACATCCTGCGAGTA -3′ | 5′-TTGGTGAGGTCAATGTCTGCTTTC -3′ |
